# Revisiting Genetic Relationships of the Endangered Austrian Turopolje With Balkan and Commercial Pig Breeds Using Genome‐Wide SNP Data

**DOI:** 10.1002/age.70104

**Published:** 2026-05-05

**Authors:** Marco Santo Cannarella, Johann Sölkner, Silvia Bruno, Elena Ciani, Gábor Mészáros

**Affiliations:** ^1^ Department of Soil, Plant and Food Sciences University of Bari Aldo Moro Bari Italy; ^2^ Department of Agricultural Sciences Universität für Bodenkultur, Wien Vienna Austria; ^3^ Department of Bioscience, Biotechnology and Environment University of Bari Aldo Moro Bari Italy

**Keywords:** Austrian Turopolje pig, genetic distances, inbreeding, local breeds, population structure

## Abstract

The endangered Austrian Turopolje (AT) pig population, which originated from six Croatian Turopolje founders imported during the early 1990s, is nowadays preserved through a national conservation project. This study aims to identify genetic relations, genetic distances and migration events between the AT population, four Austrian commercial breeds, and seven local Balkan breeds, as well as their inbreeding levels by using medium‐density single nucleotide polymorphism (SNP) arrays. The AT population was well separated from the other breeds (multi‐dimensional scaling and Admixture analyses), although displaying a relative genetic proximity to Croatian Turopolje, Hungarian Mangalitsa, and Swallow bellied Mangalitsa. AT did not show any sign of possible gene flow deriving from the other breeds, except for the Banija Spotted (Treemix analysis). The AT genetic variability did not appear negatively influenced by its recent demographic history (inbreeding based on runs of homozygosity, *F*_ROHav_ = 0.17 ± 0.05). Its inbreeding trend, although increasing, does not appear to be alarming compared to Croatian Turopolje. In conclusion, the results of this study possibly highlight how an efficient management of an originally very narrow genetic stock may have contributed to shape the current genetic make‐up of this recently established population. The observed differentiation between AT and its ancestral counterpart (Croatian Turopolje) may not only reflect the consequence of the initial founder effect but also derive from different management practices over the last three decades. This study represents a first step towards awareness raising about the originality of a local pig population traditionally characterised by the intimate connection with its rural breeding area.

## 1 Introduction

Turopolje is an autochthonous Croatian pig breed originated from past crossbreeding among the Šiška, Krškopolje, and Berkshire pig breeds (Lukić et al. 2020). After a demographic decline experienced during the Yugoslavian war and the following decade, the breed has recently regained relevance for its role in the Croatian environmental and cultural heritage (Đikić et al. 2003). In Austria, the current Turopolje population derives from a founder effect originally involving an overall of six Croatian individuals, introduced in the country in two waves, in the 1990s and 2000s, respectively (Druml et al. 2012). Specifically, in 1993, two Turopolje boars and two Turopolje sows from a single herd (ARCHE Austria 2023) were rescued during the Yugoslavian war in an attempt to save this breed from extinction (Baumung and Prevost 2010). Then, in 2000, two other boars were transferred from Croatia to Austria, likely to replenish the existing population (Baumung and Prevost 2010). Since 2001, ARCHE Austria, an association involved in the conservation of rare and endangered livestock breeds, has been appointed as the breeding organization responsible for the in situ conservation of the Austrian Turopolje (AT) herds (Baumung and Prevost 2010). In addition, the Austrian government supports the farmers who decide to contribute to the conservation of endangered breeds, including the AT pigs, through the rural development support program (ÖPUL) (ARCHE Austria 2023). In 2015, 93 breeding animals belonging to the AT population were reported (Austrian National Association for Gene Conservation 2015). Preserving the AT pig contributes to agricultural biodiversity and resilient, low‐input farming systems, as the breed is well adapted to extensive outdoor conditions typical of Austrian floodplain and woodland landscapes. Moreover, safeguarding this small population helps maintain unique genetic diversity that may be crucial for future adaptation to environmental change and supports the development of sustainable rural economies and high‐value regional products (Škorput et al. 2024).

Based on pedigree data, the AT population is supposed to be characterised by a high inbreeding level and a low genetic variability, since it derives from a small group of founders (Baumung and Prevost 2010). Consistently, Druml et al. (2012), by using a set of 25 microsatellite markers, found that the AT population has the highest values of inbreeding (*F*_IS_) and among the lowest allele richness values (RMNA) compared to the Croatian Turopolje, the Austrian Mangalitsa, the Serbian Mangalitsa, the Black Slavonian, the Pietrain, and a Bosnian mountain pig population. The study also highlighted that the AT is genetically distinct from the aforementioned pig groups, suggesting a poor gene flow among these populations. Despite the recognised conservation importance of the Turopolje pig and the documented genetic subdivision between Croatian and Austrian populations (Druml et al. 2012), there is a clear and substantial lack of phenotypic evidence supporting differentiation between these subpopulations. Peer‐reviewed studies provide detailed descriptions of morphological characteristics and growth performance for Croatian Turopolje pigs or for the breed as a whole (Karolyi et al. 2018; Škorput et al. 2024), but none of these investigations report data separately for the Austrian population. Similarly, reproductive parameters such as litter size, age at first farrowing, piglet survival and growth traits have been quantified exclusively in Croatian herds managed under traditional production systems (Karolyi et al. 2019), while no corresponding performance data are available for AT pigs. Studies addressing carcass composition and tissue distribution consistently describe the Turopolje pig as a fatty, slow‐growing breed with low lean meat content, based solely on Croatian material (Đikić et al. 2003).

To the best of our knowledge, there is currently no study investigating the genetic diversity and relationships of the AT pig population using genome‐wide single nucleotide polymorphism (SNP) markers. Thus, the aim of this work is to characterise the genetic background of the AT pig population in relation to a set of pig breeds, including four commercial breeds commonly farmed in Austria (Pietrain, Large White, Landrace, and Duroc) as well as seven Balkan autochthonous breeds (Krškopolje from Slovenia; Black Slavonian, Croatian Turopolje, and Banija Spotted from Croatia; Moravka and Swallow bellied Mangalitsa from Serbia; Hungarian Mangalitsa from Hungary) by using medium density SNP arrays. In addition, we aimed at monitoring population molecular inbreeding extent and discriminating between recent and remote inbreeding events by estimating runs of homozygosity (ROH) parameters.

## 2 Materials and Methods

### 2.1 Samples and Genotypes

**FIGURE 1:**
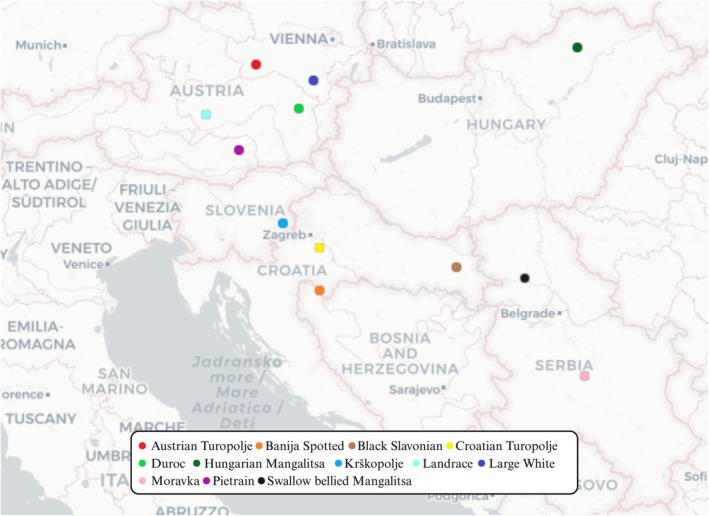
Map of geographical origin of the 12 pig populations.

**FIGURE 2:**
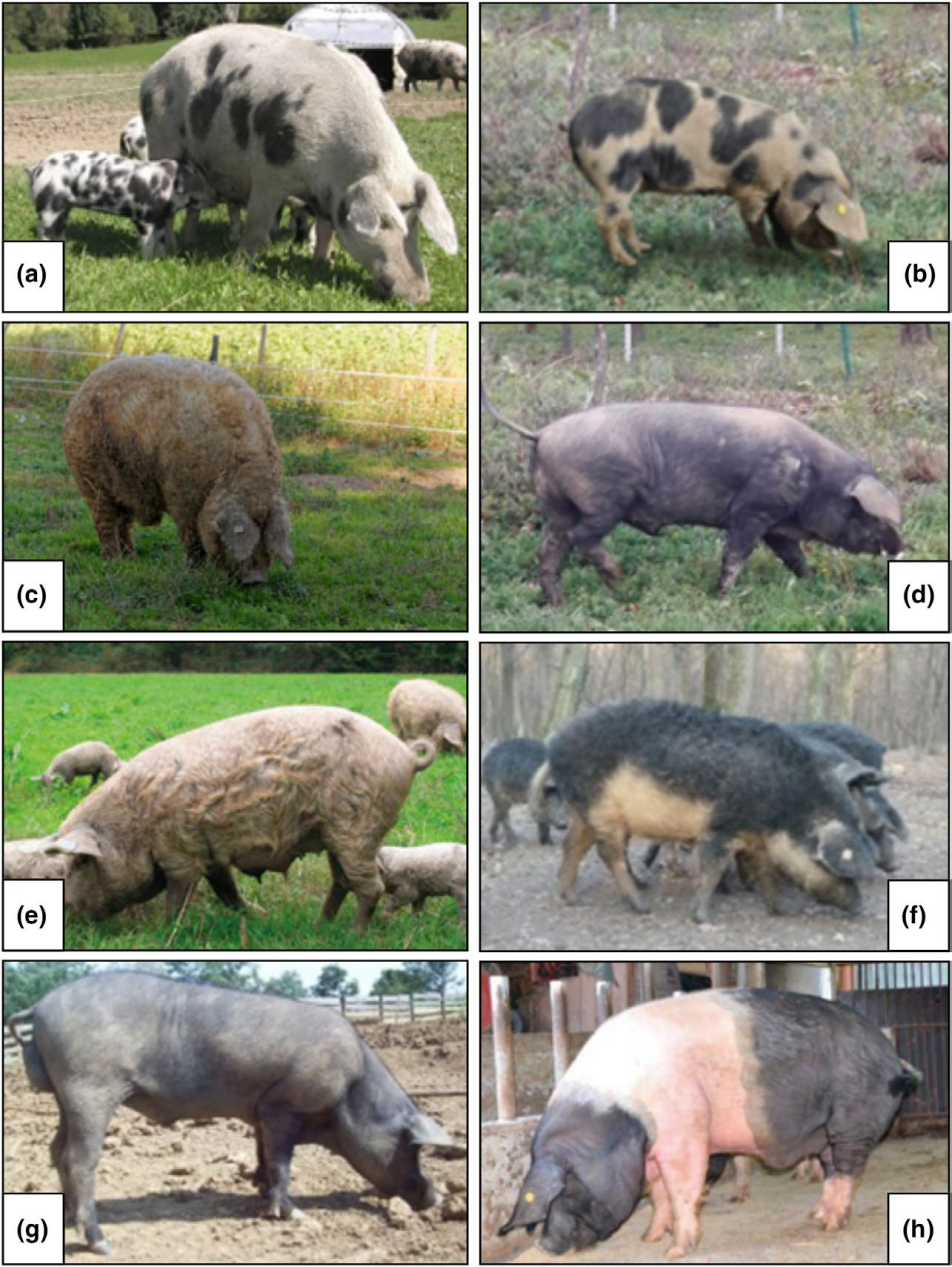
Morphological appearance of the Austrian Turopolje pigs and the seven Balkan pig breeds. (a) Austrian Turopolje (ARCHE Austria 2023); (b) Banija Spotted (Zorc et al. 2022); (c) Hungarian Mangalitsa (Addo and Jung 2022); (d) Black Slavonian (Margeta et al. 2019); (e) Croatian Turopolje (Karolyi et al. 2019); (f) Swallow bellied Mangalitsa (Agricultural Institute of Slovenia 2015); (g) Moravka (Agricultural Institute of Slovenia 2015); (h) Krškopolje (Batorek Lukač et al. 2019).

The AT genotype data were obtained, in the framework of the rural development support program (ÖPUL) run by the Austrian National Association for Gene Conservation (ÖNGENE), on a random subset of breeding animals using the *Illumina PorcineSNP60 v2 Genotyping BeadChip* (Chip 1–64 232 SNPs) developed by Ramos et al. (2009). For the commercial breeds (Pietrain—PT, Large White—LW, Landrace—LN, and Duroc—DC), animals were sampled from the Austrian national breeding programs, run by Pig Austria (pig.at). Subsets of 70–82 animals per breed, included in the routine single step genomic prediction (ssGBLUP) procedures of the breeding programs, were randomly selected from a large pool of individuals genotyped using Chip 1. The genotype dataset including Croatian Turopolje (CT), Hungarian Mangalitsa (HM) and Black Slavonian (BS) derives from the study of Lukić et al. (2020), who also employed Chip 1; the Banija Spotted (BJ) genetic data were extracted from the study of Zorc et al. (2022), obtained with the *GeneSeek Genomic Profiler (GGP) Porcine 80 K Bead Chip* (Neogen, Lincoln, NE, USA), while the dataset including Swallow bellied Mangalitsa (SBM), Moravka (MO), and Krškopolje (KP) was retrieved from Muñoz et al. (2019), whose individuals were genotyped with the *GeneSeek SNP70 BeadChip* (Muñoz et al. 2019). For every population the number of individuals and the used array formats are shown in Table S1, while their geographical origin is shown in Figure 1. The morphological appearance of the AT and Balkan breeds are depicted in Figure 2. Since sample‐size disparity (Table S1) may affect in some extent the results of the analyses, we also arranged the sample set in a randomly‐extracted balanced subset for the downstream ADMIXTURE analyses (see section “Population structure and genetic distances”). In fact, especially model‐based genetic clustering softwares tend to maximize the variance explained by clusters (Lawson et al. 2018). Larger populations could dominate the estimation of allele frequencies and have a disproportionate influence on the model, by erroneously grouping together or describing as “admixed” underrepresented populations and artificially splitting overrepresented populations into multiple clusters (Meirmans 2019).

Only markers that were common among array formats were retained in the final raw file. Notably, these were identified comparing the .*ped* files using *R v.4.2.2* (R Core Team 2022). The resulting list of variants was then used to extract the corresponding SNP genotypes from the merged dataset. The overall merged dataset included 710 pigs and 72 568 markers, out of which 32 563 were overlapping among array formats.

### 2.2 Quality Control

The merged dataset containing only the overlapping markers was subjected to filtering using *PLINK* v1.90 software (Chang et al. 2015). A SNPs missingness threshold of 0.1 (*geno 0.1*), an individual missingness threshold of 0.1 (*mind 0.1*), and a minor allele frequency threshold of 0.02 (*maf 0.02*) were considered. These parameter values were selected based on previous research conducted on pigs (Banos et al. 2022; Lemus‐Flores et al. 2023; Schiavo et al. 2021), resulting in the initial dataset (I_D), composed of 706 individuals and 32 114 variants. Filtering criteria applied to I_D to obtain the datasets used in the current study, according to the intended use, are described in Table S2.

### 2.3 Population Structure and Genetic Distances

A multi‐dimensional scaling (MDS) analysis was conducted, using PLINK v1.90, on three different datasets to focus the attention on the AT population. Aside from the I_D, two reduced datasets were obtained subsequently removing the following breeds: (i) Large White, Pietrain, Duroc and Landrace (MDS_NC_D); (ii) Croatian Turopolje, Hungarian Mangalitsa and Swallow bellied Mangalitsa (MDS_R_D).

To infer the population structure, four admixture analyses were performed on as many datasets: *unbalanced unsupervised* (UB_US_D), *balanced unsupervised* (B_US_D), *unbalanced supervised* (UB_S_D), and *balanced supervised* (B_S_D), where “balanced” refers to the sample size of each population, while “supervised” refers to the assignment of an a priori information about the group membership to every individual except the ones belonging to the population of interest. The balanced datasets were obtained randomly selecting 20 individuals from each population, to address the presence of biases due to sample‐size disparity. *ADMIXTURE 1.3.0* (Alexander et al. 2009) was used to carry out the aforementioned analyses. In order to assure using only independent loci, the admixture analyses were performed after pruning based on linkage disequilibrium between marker pairs (*−‐indep‐pairwise*). A window size of 50 bp, a step size of 5 markers, and an *r* ^2^ parameter of 0.5 were adopted. The best number of clusters (K) to be retained in *ADMIXTURE* was estimated via cross‐validation (CV), and the model with minimum CV error was selected. The results were double‐checked via the EVANNO approach, based on the differences between CV errors of two consecutive K values (Evanno et al. 2005). AT individuals with a major membership component (qi) lower than 0.8 were arbitrarily defined as admixed. To further investigate the population structure, a *Mutual Nearest Neighbor network* (*MNN network*) analysis was performed using the *R package NetView* (Neuditschko et al. 2012). For this analysis, as suggested in Neuditschko et al. 2012, markers with extreme deviation from Hardy Weinberg Equilibrium, considering a *p* < 0.001, were excluded using PLINK v.1.90, resulting in a specific dataset (MNN_D). Unlike in the admixture analysis, the K value refers to the maximum number of nearest neighbors that the software infers for every individual. This process leads to the formation of nets interconnecting the different individuals according to their genetic relationship. The analysis was performed adopting K values ranging from 1 to 20.

The *F*_st_ index describes the proportion of the total genetic variance contained in a subpopulation, relative to the total genetic variance (Wright 1984). The higher the index, the higher the genetic distance among populations (Wright 1984).

$$F_{\mathrm{ST}}=\frac{H_{T}-H_{S}}{H_{T}}$$

where *H*_T_ is the expected heterozygosity in the entire population, while *H*_S_ is the expected heterozygosity in the single subpopulation. Pairwise *F*_ST_ indexes were computed with the *R package StAMPP* (Pembleton et al. 2013). For this analysis, the dataset was filtered only for SNP missingness (*geno 0.1*) and individual missingness (*mind 0.1*) (GD_I_D). In fact, the use of the *‐‐maf* function would delete the majority of fixed (or almost fixed) alleles, providing a genetic distance lower than the real one, therefore it was not used for this calculation.

The same dataset and *R package* were also used to calculate the Nei's distances. The difference between the *F*_ST_ and Nei's distances lies in the type of evolutionary forces that influence the allelic frequency. The former considers only random drift as an evolutionary force, while the latter also considers the influence of mutation; therefore, these two distances allow us to observe divergence events respectively more and less recent. Finally, two neighbor networks, based on the two types of distances, were constructed and plotted using *SplitsTree v.4.14.2* (Huson and Bryant 2006).

### 2.4 Inference of Migration Events

To assess migration events, we tested the I_D with *Treemix v.1.1.12* (Pickrell and Pritchard 2012), adopting the default parameters, except for the *‐k* argument where a window size for the LD of 100 markers (*−k 100*) was considered, to account for the fact that nearby SNPs are not independent. Moreover, we used the *R package OptM* to infer the best value of *m* (number of migration events). The *f4* test, implemented in the *Treemix* software, was performed to test the validity of a four‐population ((A, B) (C, D)) phylogenetic tree. In particular, a significant deviation of the *Z*‐score values from 0 is related to the presence of gene flow in the tree, resulting in a phylogeny not completely tree‐like. A significantly positive *Z*‐score indicates gene flow between populations related to either A and C or B and D and a significantly negative *Z*‐score indicates gene flow between populations related to A and D or B and C.

### 2.5 Inbreeding, ROHs and Heterozygosity

Individual inbreeding coefficients (*F*_IS_) were estimated for each single locus of I_D, using the *‐‐het* command in PLINK v.1.90. In addition, runs of homozygosity were computed on the GD_I_D according to the default parameters, except for the minimum SNPs density (‐‐*homozyg‐density 100*) and the minimum number of SNPs (*‐‐homozyg‐snp 15*). Indeed, for these parameters we decided to adopt more relaxed thresholds in order to account for the dataset's SNPs density.

ROHs were sorted into four arbitrary groups, according to the following ranges of length: 2–4 Mb, 4–8 Mb, 8–16 Mb, and > 16 Mb. This step was essential to infer the age of inbreeding for each population. In fact, a higher number of large ROHs in the genome can be linked to recent inbreeding because the crossing‐over events have not had enough time to reduce the length of the ROHs (Schiavo et al. 2021). The overall coefficient of inbreeding based on ROHs (*F*_ROH_), and the *F*_ROH_ for all window sizes and thresholds were computed dividing the sum of the identified ROHs for an individual by the total length of the autosomal genome of *Sus scrofa* (2265.77 Mb) (Warr et al. 2020), according to the following formula (McQuillan et al. 2008)

$$F_{\mathrm{ROH}}=\frac{\sum L_{\mathrm{ROH}}}{L_{\text{auto}}}$$

in which ∑*L*_*ROH*_ is the total length of all of an individual's ROHs above a specified minimum length and *L*_auto_ is the length of the autosomal genome covered by SNPs, excluding the centromeres.

In parallel, runs of homozygosity were also identified using the R package *detectRUNS* (Biscarini et al. 2018), which works according to the method of “consecutive SNPs” unlike the “sliding‐window” method used by *PLINK*. The package calls a ROH if the genomic segment is longer than a minimum threshold (1000 Kb) and if there are no heterozygous markers in it; therefore, it does not account for the presence of a minimum number of polymorphic sites in the ROH, in contrast to the “sliding‐window” method (where 1 heterozygous genotype every 1000 Kb window is allowed).

The intra‐population genetic variability was also assessed through the heterozygosity analysis, performed with the *R package BITE* (Milanesi et al. 2017) on the same dataset used for the inbreeding level assessments.

## 3 Results

### 3.1 MDS Analysis

**FIGURE 3:**
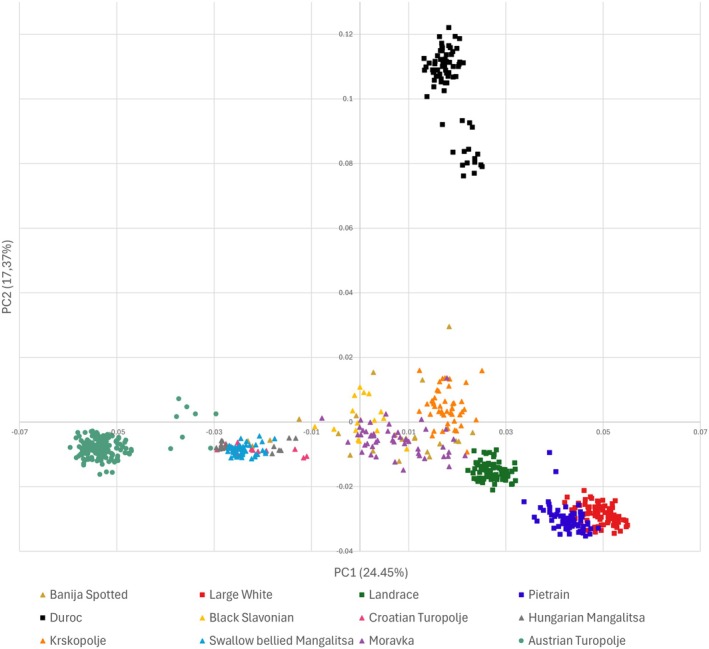
Population structure, analysed with a Multi‐Dimensional Scaling (MDS), of the 12 considered pig populations. AT population, Balkan breeds and commercial breeds are respectively marked with dots, triangles and squares.

The MDS plot carried out on the overall dataset showed a good breed separation (Figure 3), with pigs from the Balkans (BJ, MO, SBM, BS, CT, HM, KP) in between the AT population and the Austrian commercial breeds. In particular, the Balkan breeds were divided into two groups, one (including Black Slavonian, Banija Spotted, Moravka and Krškopolje) nearer to the commercial breeds and the other (including Hungarian Mangalitsa, Croatian Turopolje, and Swallow bellied Mangalitsa) nearer to the AT population. In contrast to the expectations, only few individuals overlapped between Croatian and AT populations, indicating a relatively high degree of differentiation between the two groups.

To further test the robustness of the observed relationship pattern, another MDS analysis was carried out considering the MDS_NC_D (Figure S1a). In this case, the AT population appeared more differentiated from Hungarian Mangalitsa, Croatian Turopolje, and Swallow bellied Mangalitsa, while being closer to the Banija Spotted. The genetic relationship between AT and Banija Spotted was also confirmed through a MDS analysis conducted on the MDS_R_D (Figure S1b).

### 3.2 Admixture Analysis

**FIGURE 4:**
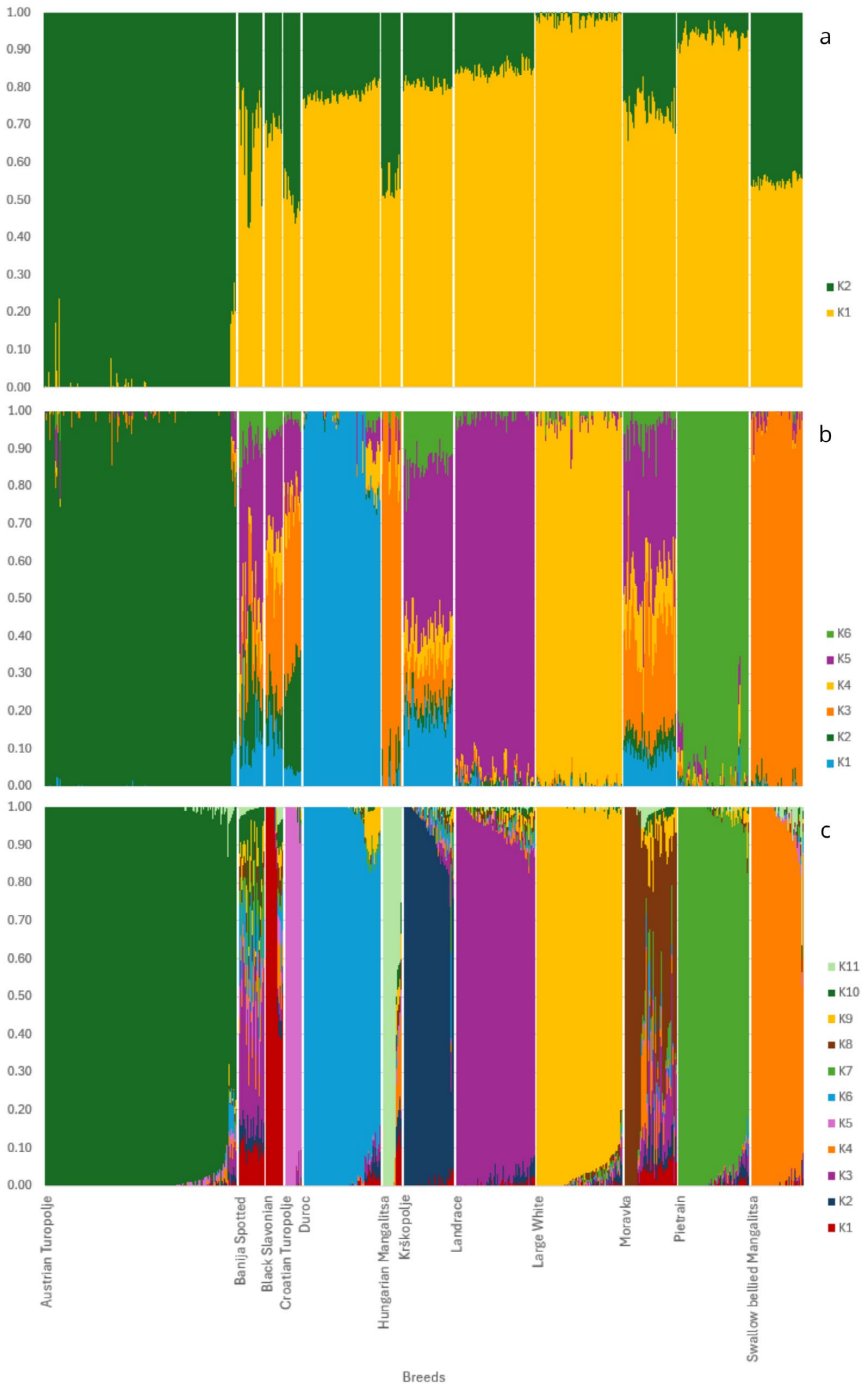
Analyses performed on ADMIXTURE 1.3.0. Every vertical line refers to an individual and is divided into K segments. The segment length is proportional to the genetic component belonging to a specific cluster. Here there are the main significant K values for the UB/US analyses: K2 (a), K6 (b) and K11 (c).

The unbalanced and unsupervised admixture analysis (UB/US) performed on the LD‐pruned dataset (containing 19 891 loci), at *K* = 2 (CV error: 0.65) supported the MDS results, showing that AT is genetically separated from the Austrian commercial populations. Moreover, the Balkan breeds were characterised by the presence of both components (i.e., the commercial one and the one related to AT), suggesting that they have preserved some autochthonous genetic material, despite the documented past crossbreeding events with commercial breeds (Figure 4a). At *K* = 6 (CV error = 0.56), the four commercial breeds and the AT had each their own cluster. Swallow bellied Mangalitsa and Hungarian Mangalitsa breeds clustered together. The remaining Balkan breeds (BJ, MO, BS, CT, KP) were highly admixed, clearly showing both a commercial and a local genetic background (Figure 4b).

According to the model with minimum CV error, and to the EVANNO model (Figure S2), the best value of *K* was detected at *K* = 11 (CV error = 0.53) (Figure 4c). At this value, almost every breed had its own cluster, with some exceptions such as (i) the Banija Spotted, which appeared highly admixed (with a major genomic component belonging to Landrace and Black Slavonian), (ii) the Moravka, which showed a prevalence of Landrace and Swallow bellied Mangalitsa components, and, to a lesser extent, (iii) the Black Slavonian and the Hungarian Mangalitsa, showing only a few admixed individuals. Similarly, the admixture analysis highlighted the presence of few admixed individuals within the AT population (the admixture mainly concerning the Duroc and the Landrace breeds), as also shown in the MDS plot. Overall, the number of admixed AT individuals was 22 out of 184 total individuals (11.96%) (Table S3).

The results of B/US analysis at *K* = 11 seemed to be very similar to the ones observed in the UB/US analysis (Figure S3a). On the contrary, the results at *K* = 2 highlighted a relevant presence of a genetic component mainly ascribable to the commercial breeds in the AT population (Figure S3b).

**FIGURE 5:**
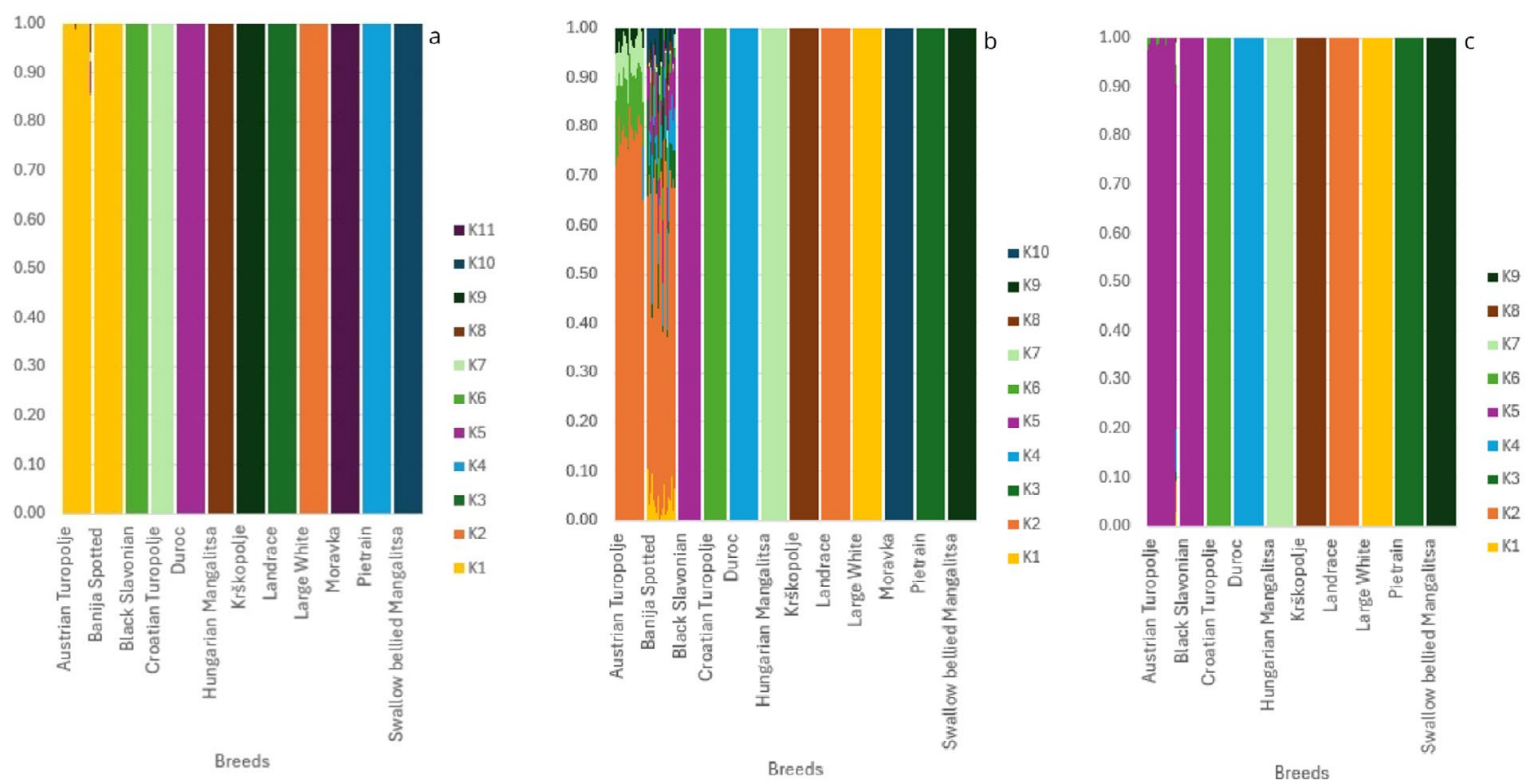
Analyses performed on ADMIXTURE 1.3.0. Every vertical line refers to an individual and is divided into K segments. The segment length is proportional to the genetic component belonging to a specific cluster. Here are depicted the results of B/S ADMIXTURE analyses, carried out on the B_S_D providing a priori information about group membership to all the breeds but the AT (a), and to all the breeds but AT and BJ (b). Finally, it is depicted the B/S admixture plot obtained on the R_B_S_D providing a priori information about group membership to all the breeds but the AT (c).

The genetic relationship observed in the MDS plot between AT and Banija Spotted was also supported by the balanced supervised (B/S) ADMIXTURE analysis when carried out providing a priori information about group membership to all the breeds but the AT (Figure 5a). Since the above result may be affected by the high level of admixture of the Banija Spotted, we first performed a B/S analysis without specifying the population data either for AT individuals or for Banija Spotted ones. In this case, both population samples showed a major genetic component belonging to Landrace, a phenomenon possibly linked to a “dragging effect” (see the Discussion section) over AT, mediated by the highly admixed Banija Spotted (Figure 5b). Then, we performed a B/S analysis on a dataset devoid of the strongly admixed breeds (BJ and MO) (R_B_S_D), thus highlighting that the AT population shared now its major genetic component with the Black Slavonian (Figure 5c).

### 3.3 Mutual Nearest Neighbour Network Analysis

**FIGURE 6:**
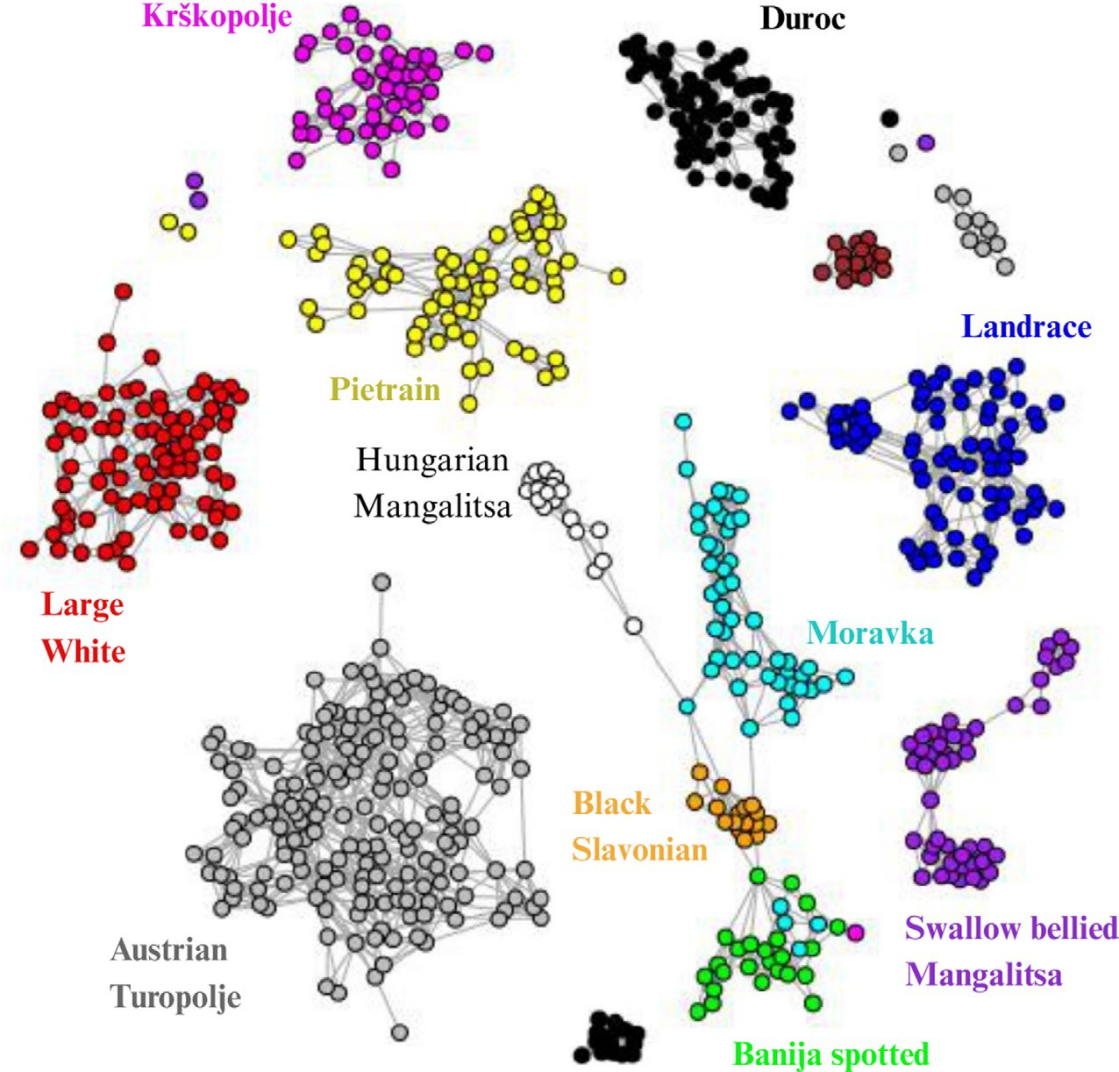
Mutual Nearest Neighbor network at *K* = 12, performed with the R package Netview. Each dot represents an individual, whose colour is referred to the belonging population.

Similarly, to the MDS and admixture analyses, the MNN network analysis, at an arbitrarily chosen value of *K* = 12 (Figure 6), depicted connections mainly among Banija Spotted, Moravka, and Black Slavonian. Unlike the admixture analysis, the CT and the KP breeds appeared here each clearly separated from the rest of the breeds. A connection between the group of the above Balkan breeds (BJ, MO, BS) and the AT was observed at *K* = 19 (Figure S4). This linkage was mediated by a subset of seven AT individuals, five of which were included in the list of the AT individuals previously highlighted as admixed.

### 3.4 Analysis of Genetic Distances

**FIGURE 7:**
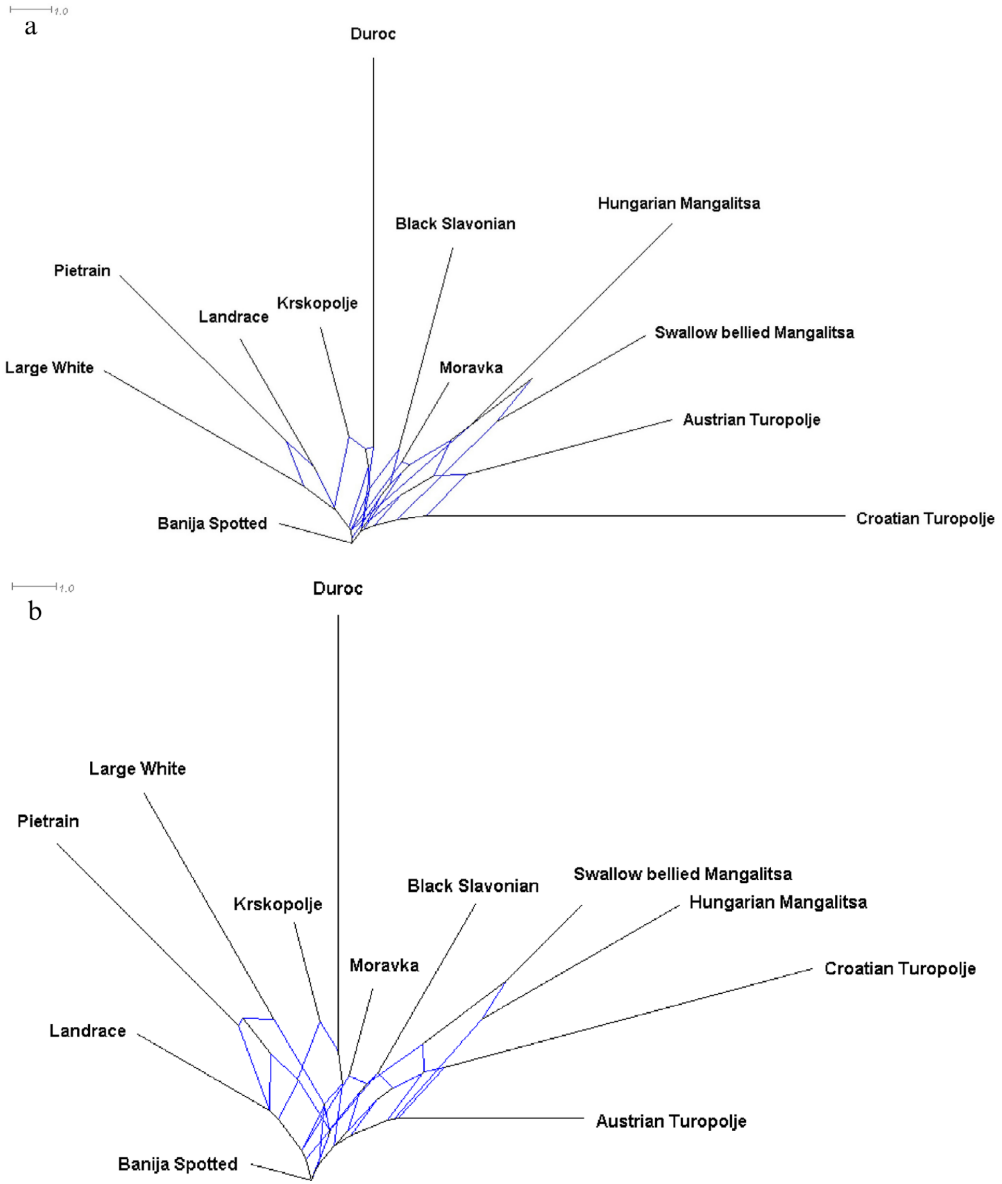
Cluster networks based on FST (a) and Nei's (b) distances computed on the GD_I_D.

Genetic distances were inferred considering a dataset of 706 individuals and 32 148 markers (i.e., without filtering for the ‐‐*maf* parameter). The pairwise *F*_ST_ indexes of Wright (Table S4), showed value ranges (0.08–0.35) that were consistent with those previously reported in the literature for the considered breeds. In accordance with the admixture and the MDS results, the Banija Spotted breed appeared to be generally the closest to the other breeds in the dataset. The AT *F*_ST_ indexes ranged between 0.14 and 0.26 (with Banija Spotted and Duroc, respectively). The Nei's distances followed the same pattern described by the *F*_ST_ (Mantel test correlation index: 0.91; *p*‐value: 10^−4^), with some exceptions represented by the less extreme values of Croatian Turopolje and Hungarian Mangalitsa (Table S4) when contrasted to the other breeds. The networks based on *F*_ST_ (Figure 7a) and Nei's (Figure 7b) distances describe the genetic connections among the considered breeds, placing the BJ breed as the nearest to the root and the DC as the furthest. In addition, both networks locate the AT population close to the Croatian Turopolje, the Swallow bellied Mangalitsa and the Hungarian Mangalitsa To further test the robustness of the *F*_ST_ distances observed between the Balkan breeds and the AT population, a new network was created removing the commercial breeds from the dataset. It resulted that the relationships' patterns between the AT population and the three aforementioned local Balkan breeds remained unchanged (Figure S5).

### 3.5 Migration Events

**FIGURE 8:**
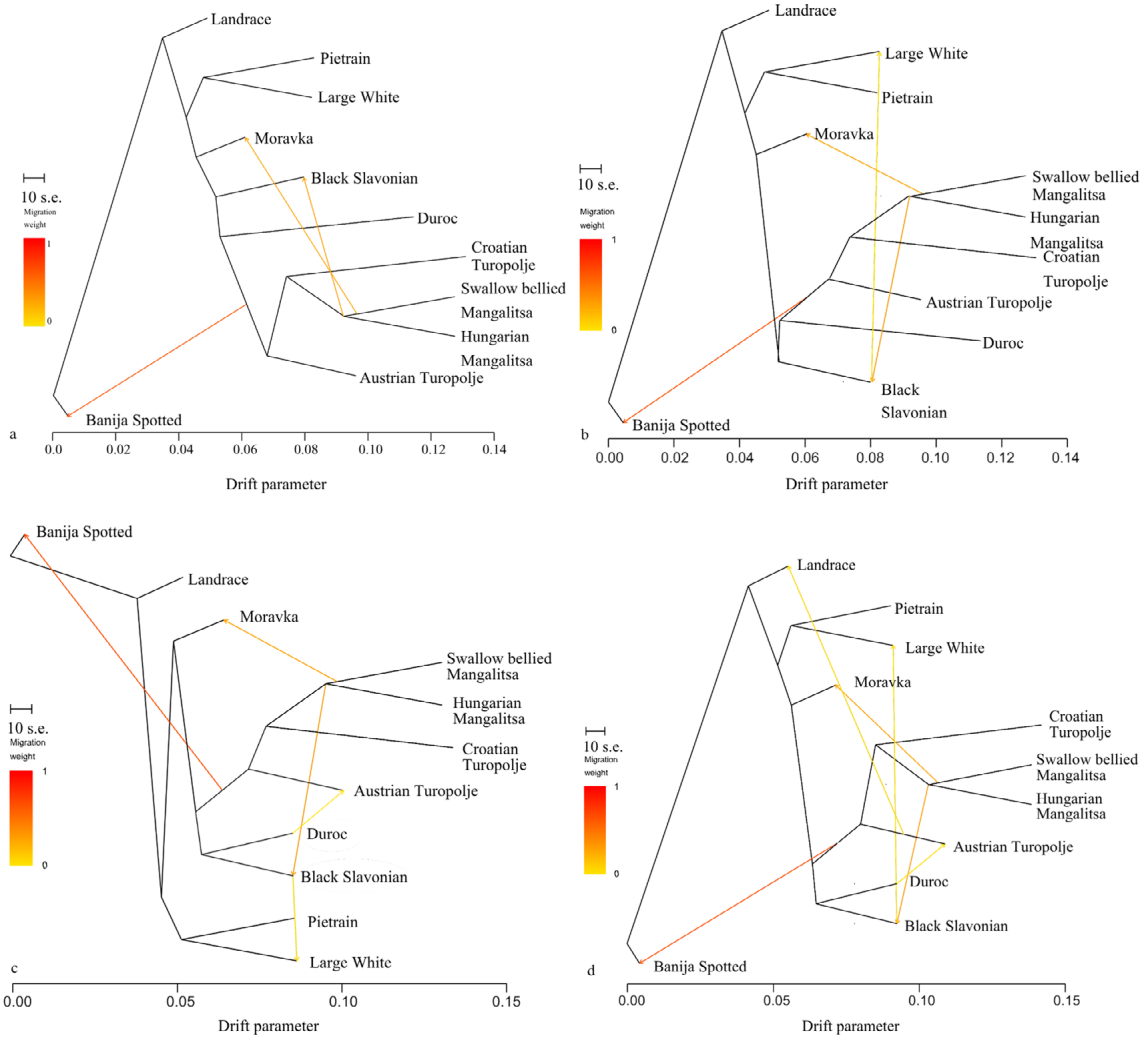
The OptM plot (a), obtained with the R package OptM, for *m* (number of migration edges) ranging from 0 to 15. The white dots represent the different values of *m*, the stars represent the changing points, while the differently coloured lines represent the four considered inferring models. Only at *m* = 3 two out of four change points (Piecewise Linear and Bent Cable) resulted to be overlapped. TreeMix estimation of phylogenetic network and relationships among pig populations with three migration events (b).

As shown in Table S5, the software *Treemix* highlighted, for *m* (number of migration edges) from 0 to 15, the presence of several migration events among the breeds in the dataset. According to the *R package OptM*, the best value of *m* ranged from 3 to 6, depending on the considered model (Figure 8a). Based on the superimposition of the change points only at *m* = 3 for two (Piecewise Linear and Bent Cable) out of the four models, we choose to display this *m* value (Figure 8b). The results for the above m values showed migration flows between the local Balkan breeds, particularly between. The tree‐topology highlighted, consistently with previous analyses, a group of Balkan breeds clustered together (CT, SBM, HM, BJ, and AT). The positioning of the remaining Balkan breeds (BS, KP, and MO) suggested a possible influence of commercial breeds. Notably BS displayed a proximity with Duroc, Moravka and KP clustered with the branch including the commercial breeds LW, LN, and PT. The three migration events, in order of inferred extent, concerned LN and BJ (edge weight range: 0.4–0.6), the Croatian Turopolje‐Mangalitsa branch and BS (edge weight range: 0.4–0.6), and between the Croatian Turopolje‐Mangalitsa branch and MO (edge weight range: 0.2–0.4), as shown in Figure 8b. Also when considering the results from *m* = 0 to *m* = 15, these three migration events appeared to be the most supported (almost 100% of tested instances).

### 3.6 F4 Test

For the AT, the Treemix *fourpop* analysis, at the highest *Z*‐score values (≥ 50), highlighted the highest numbers of migration occurrences with the CT (32), SBM (25), and HM (25) Balkan breeds. The same pattern was displayed when considering *Z*‐score values ≤ −50, thus corroborating the results observed in the MDS and in the distance networks analyses (Table S6). In addition, a notable number of migration occurrences was reported between AT and the commercial DC population (26), as shown in Figure 8c,d.

### 3.7 Identification of ROHs, Inbreeding Levels and Heterozygosity

Individual inbreeding coefficients (*F*_IS_) and average *F*_IS_, for each breed, are shown in Table S7. The lowest value was observed in Landrace (*F*_IS_ = 0.06 ± 0.03), while the highest values were related to Croatian Turopolje (*F*_IS_ = 0.46 ± 0.11) and Hungarian Mangalitsa (*F*_IS_ = 0.56 ± 0.14).

**FIGURE 9:**
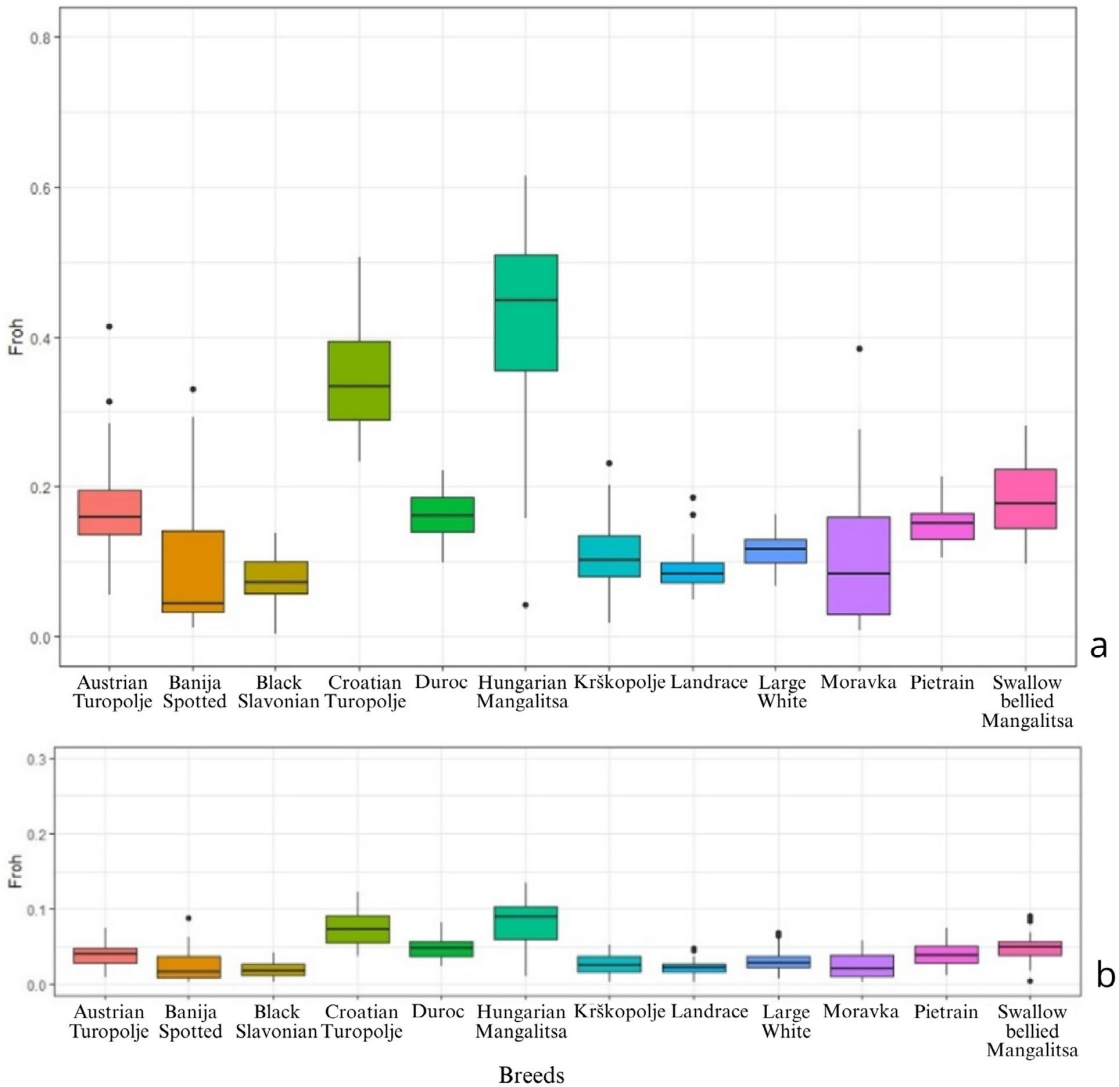
Inbreeding coefficients related to every analysed breed. Total FROH related to ROHs identified with PLINK 1.9 (a), total FROH related to ROHs with a length ranging between 8 and 16 Mb (b).

The average number of ROHs (*N*_SEG_), the average segment length (*L*_ROH_), the average *F*_ROH(> 1Mb)_ values, and the average heterozygosity (HETobs) for the considered populations are shown in Table 1, while in Figure 9a the boxplots for the inbreeding coefficients based on ROHs > 1 Mb are reported.

**TABLE 1:** ROHs parameters related to every considered breed.

Breeds	*N*_SEG_ (SD)	*L*_ROH_ (SD)	*F*_ROHav_ (SD)	HET_obs_ (SD)
Austrian Turopolje	34 (6.6)	11.11 (2.4)	0.17 (0.05)	0.29 (0.02)
Banija Spotted	24 (11.5)	8.34 (3.9)	0.09 (0.09)	0.35 (0.05)
Black Slavonian	16 (5.8)	10.41 (3.8)	0.08 (0.04)	0.34 (0.02)
Croatian Turopolje	55 (13.4)	14.44 (2.7)	0.35 (0.08)	0.20 (0.04)
Duroc	51 (6.6)	7.11 (0.8)	0.16 (0.03)	0.31 (0.02)
Hungarian Mangalitsa	57 (15.7)	15.85 (4.3)	0.41 (0.14)	0.17 (0.06)
Krškopolje	23 (5.0)	10.36 (2.9)	0.11 (0.04)	0.35 (0.02)
Landrace	30 (5.5)	6.53 (1.1)	0.09 (0.02)	0.36 (0.01)
Large White	40 (6.0)	6.58 (0.9)	0.12 (0.02)	0.35 (0.01)
Moravka	19 (9.8)	10.53 (5.4)	0.11 (0.09)	0.34 (0.04)
Pietrain	41 (6.3)	8.28 (1.2)	0.15 (0.03)	0.34 (0.02)
Swallow bellied Mangalitsa	39 (6.9)	10.84 (2.5)	0.19 (0.05)	0.24 (0.02)

*Note:* *N*_SEG_: Average number of the ROHs and standard deviation (SD) for each breed. *L*_ROH_: Average length of the ROHs (in Mb) and standard deviation (SD) for each breed. *F*_ROHav_: Average inbreeding coefficient and standard deviation (SD) for each breed. HET_obs_: Average observed heterozygosity and standard deviation (SD) for each breed.

Black Slavonian and Hungarian Mangalitsa showed the lowest (16) and the highest (57) *N*_SEG_ values, respectively. Landrace and Large White displayed the lowest *L*_SEG_ (6.53 and 6.58 Mb, respectively), while the Hungarian Mangalitsa showed the highest *L*_SEG_ (15.85 Mb). As for the average *F*_ROH_ values, the lowest value was observed in the Black Slavonian (*F*_ROHav_ = 0.08 ± 0.04), while the highest values were observed in the Hungarian Mangalitsa (*F*_ROHav_ = 0.41 ± 0.14) and the Croatian Turopolje (*F*_ROHav_ = 0.35 ± 0.08), in accordance with the average *F*_IS_ values. The overall *F*_ROH_ results are consistent with the results obtained using the R package *detectRUNS* (*r* ^2^_PLINK/*R*_ = 0.95) and with previous studies conducted on the same Balkan populations (Lukić et al. 2020; Schiavo et al. 2020; Zorc et al. 2022). The boxplot based on the *F*_ROH_ for ranges 2–4 Mb, 4–8 Mb, 8–16 Mb and > 16 Mb are presented in Figure 9b. The 2–4 Mb range showed, in general, no remarkable differences among the breeds, except for slightly higher values for the commercial breeds. Considering the *F*_ROH_ ranging from 4 to 8 Mb, we observed an initial increase of inbreeding values also for CT and HM. Finally, for higher *F*_ROH_ windows (8–16 Mb and > 16 Mb), HM and CT emerged as the highest inbred populations, with a broad range of variability of the individual inbreeding coefficient's values (Table 2).

**TABLE 2:** ROHs parameters, based on different window sizes, related to AT and CT populations.

ROHs size (Mb)	Generations ago	Years ago	*F*_ROHav_ (SD) AT	*F*_ROHav_ (SD) CT
2–4	25–12	75–36	0.01 (0.004)	0.01 (0.005)
4–8	12–6	36–18	0.03 (0.009)	0.04 (0.014)
8–16	6–3	18–9	0.04 (0.015)	0.08 (0.027)
> 16	3–1	9–3	0.09 (0.045)	0.22 (0.063)

*Note:* *F*_ROHav_: Average inbreeding coefficient and standard deviation (SD) for each breed.

**FIGURE 10:**
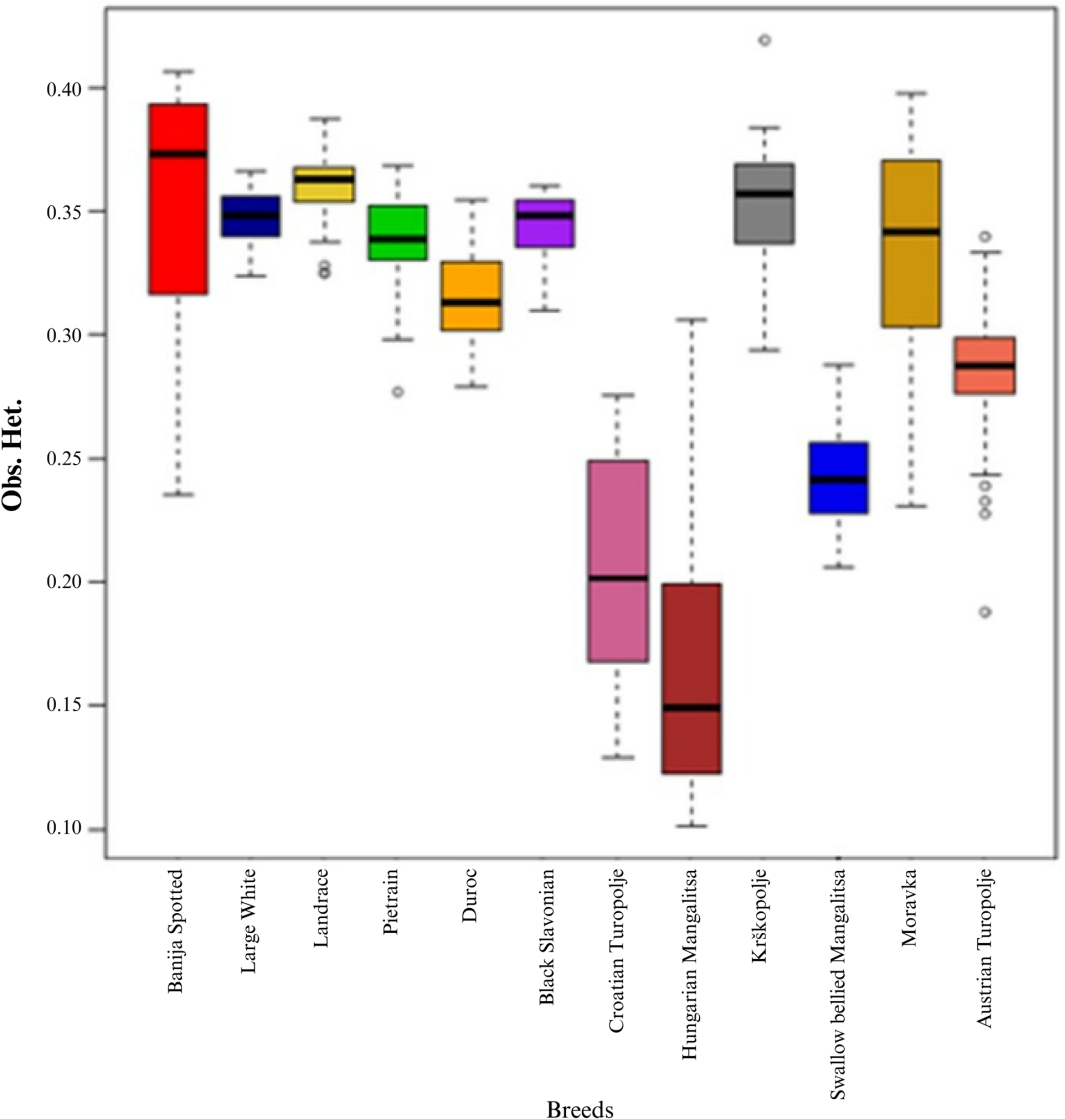
Observed heterozygosity for each breed, computed with R package BITE.

The highest percentage of individuals with an *F*_ROH_ value higher than 0.25 was observed in the Hungarian Mangalitsa (88.9%), followed by the Croatian Turopolje (87.5%). In accordance with the inbreeding results, the lowest average values of observed heterozygosity (HET_obs_) (Table 1) were associated with Hungarian Mangalitsa (HETobs = 0.17 ± 0.06), followed by Croatian Turopolje (HET_obs_ = 0.20 ± 0.04). On the contrary, the Banija Spotted had clearly higher values, while AT occupied an intermediate position (HET_obs_ = 0.29 ± 0.02), confirming the general pattern (Figure 10).

## 4 Discussion

The study of the genetic relationship highlighted separation of the AT population from the other breeds. The above emerged mainly from the MDS analysis and the unsupervised admixture analysis at *K* = 2. This separation might be due to a random drift phenomenon, either (i) starting from the establishment of the population (founder effect), (ii) caused by the subsequent reproductive isolation of the individuals, or (iii) owing to a synergic effect of the two former events. Another hypothesis could hinge on the introgression of genetic material into the AT population from breeds not considered in the dataset. This hypothesis seems less likely because of the lack of historical‐scientific evidence. In the current study, the AT population did not show relevant signs of gene flow deriving from the other considered populations. For instance, the AT population spatial distribution is quite dense in the MDS plot and, consistently, the observed heterozygosity levels are not very high compared to the values observed in the other populations, such as CT and HM. Despite the founder effect and the reproductive isolation mentioned above, the AT population presented a lower *F*_ROH_ (0.17 vs. 0.35) and higher observed heterozygosity values (0.29 vs. 0.20) when compared to the CT breed. This could be linked to a more severe and recent loss in genetic diversity in the CT breed, possibly due to a second bottleneck experienced after the war (Begemann 2016) and/or weaker inbreeding management approaches in CT versus AT.

The MDS analysis and the cluster networks brought out a relation of a certain genetic proximity between AT population and Croatian Turopolje, Hungarian Mangalitsa and Swallow bellied Mangalitsa breeds. The proximity between Austrian and Croatian Turopolje populations can be easily attributed to the role of the latter as origin population in recent times (Baumung and Prevost 2010), while the closeness with the other two breeds could be due to the lack of massive crossbreeding events of HM and SBM with commercial breeds (Radović et al. 2019; Kasprzyk and Walenia 2023). This lack may have resulted in the conservation of ancestral characters, possibly due to their common origin from the Serbian Šumadinka breed (Radović et al. 2019; Radnóczi 2012; Kasprzyk and Walenia 2023). Certain genetic proximity relationships have been identified, in the balanced supervised admixture analysis, for AT also with the Banija Spotted breed and the Black Slavonian breed. Similarly to the hypothesis formulated to explain the closeness of AT with CT, HM and SBM, also the closeness of AT with BJ and BS could be due to the residual presence of an ancestral genetic component, persisting in the genome of the Banija Spotted and Black Slavonian. In fact, according to Luković et al. (2023), the BJ breed has been developed in the late 19th century by crossing the local Turopolje pig with enhanced breeds, while the BS breed originated from Croatian Mangalitsa (Hrasnica 1958). The observed phenomenon, at least for AT‐BJ, is also corroborated by the Treemix topology and also phenotypically supported by the similarity of the coat pattern in the two populations The MDS analysis highlighted that some Balkan breeds, including Black Slavonian, Banija Spotted, Krškopolje, and Moravka, seem to be genetically close also to the considered commercial breeds. The above relationships could be explained by their genetic and breeding history. Indeed, as hinted at above, Black Slavonian originated by crossing Croatian Mangalitsa gilts with Berkshire boars, and then by improving the offspring with Berkshire and Large Black individuals (Hrasnica 1958). On the other side, Krškopolje was crossed with various English breeds to enhance economically relevant traits (Kastelic and Čandek‐Potokar 2013). Similarly, Moravka was crossed with Berkshire and Yorkshire at the beginning of the 20th century (Živković and Kostić 1952). Also Banija Spotted experienced crossing events with Berkshire and Landrace‐type pigs since the establishment of the breed (Luković et al. 2023). The latter gene flow (LN‐BJ) was also highlighted via a migration event inferred by the Treemix analysis. Therefore, the BJ historical background might explain both its high number of admixed individuals and its “dragging effect” over the AT population observed in the B/S ADMIXTURE analysis (Figure 5b). This phenomenon refers to the apparent attraction of a population (AT) toward another (LN) due to shared ancestry with an admixed intermediate population (BJ), rather than due to direct gene flow. In fact, the composite allele frequency structure of highly admixed populations can dominate the shared patterns that the algorithm tries to explain. This can lead to a “general” cluster that resembles the major component shared across many individuals (e.g., Landrace‐like), because the signal is statistically prominent in the data even if it is not a proper ancestral structure, as reported in ADMIXTURE simulation studies (Alexander and Lange 2011). The unsupervised admixture analysis at *K* = 11 showed the presence of a limited number of AT individuals with an admixture percentage ranging from 10% to 25%. These subjects can be identified and cautiously used in the breeding programs, aiming to the conservation and the further expansion of the AT population.

Interestingly, a more significant inbreeding seems to appear in the Croatian Turopolje population compared to the Austrian counterpart only in the last 3–6 generations (ROH size equal to 8–16 Mb), corresponding to a temporal window of ≈9–18 years ago (assuming a generation interval of about 3 years, as in Zhang et al. 2022). This outcome may be related to the most recent genetic management of the two populations. Indeed, similarly to AT, CT also experienced a recent founder effect. In the post‐war period (1996), a new herd‐book was in fact established for the CT, starting from 12 sows and only 3 boars (Begemann 2016). Despite demographic expansion having been reported for this breed in 2008 (Begemann 2016) and 2022 (Croatian Agency for Agriculture and Food 2022), no information is available, to the best of our knowledge, about the original degree of relationship among the herd‐book founders. If a high kinship was present among them, avoiding inbreeding in the subsequent generations would have been challenging.

As for AT, the inbreeding trend inferred since 25 generations ago, although increasing, does not appear to be alarming. This is probably connected to the positive effects of various projects, started during the last decade, focused on the conservation and reintroduction of local breeds. The aforementioned rural development support program (ÖPUL) is committed in this regard, preserving the Austrian local livestock breeds (ARCHE Austria 2023). This and other similar projects reflect a change of course supported, among others, by the FAO through the proposal of new policies aimed at the preservation, utilisation and improvement of indigenous livestock breeds (FAO 2007). Practical conservation strategies to preserve genetic diversity, reduce inbreeding levels, maintain breed identity and manage admixed individuals may, in future, leverage AT genotype data from high‐density SNP array, allowing finer reconstruction and inference of heterozygosity and inbreeding levels, effective population size and distribution of admixed individuals (Eusebi et al. 2020). Furthermore, a reduction of coancestry among breeding animals through the Optimum Contribution (OC) method may help to maximise the retention of the available genetic diversity. The OC method is indeed recommended for small livestock populations and consists of assigning to each breeding animal a contribution score, based on its relationship to the rest of the population, prioritising lower‐kinship individuals to reduce inbreeding (Fernández et al. 2011). In this regard, admixed AT individuals can be moderately harnessed in breeding programs, at least until the core purebred group size is small. Finally, the periodical re‐assessment of the genetic diversity, the adjustment of breeding goals based on changes in effective population size, inbreeding trends and fitness traits could help defining an adaptive management system in the long‐term (Tăpăloagă et al. 2025). Another management option, able to enhance within‐population genetic diversity and mitigate the negative effects of inbreeding, especially for CT, may be the planning of a carefully controlled crossbreeding plan between the Austrian and Croatian Turopolje populations, particularly in light of founder events and demographic bottlenecks experienced by both populations. Controlled gene flow may indeed increase heterozygosity and help empower fitness potential (Kristensen et al. 2015; Hoffmann et al. 2020). However, such intervention must be applied cautiously, as inappropriate crosses may result in outbreeding depression or compromise breed identity (Weeks et al. 2011). Minimizing these risks requires rigorous genomic and phenotypic monitoring to ensure a balance between genetic enrichment and local adaptation.

In conclusion, the AT population appeared to be genetically sufficiently separated to be possibly identified as a distinct breed in the future. Moreover, the AT genetic variability deserves further investigation as it may harbour variants useful for traits of economic interest. Finally, the proximity of AT with breeds characterised by ancestral traits and its ability to thrive in extensive farming systems could contribute to the conservation of traditional Austrian agro‐ecological landscapes.

## Author Contributions

**Marco Santo Cannarella:** formal analysis, investigation, visualization, writing – original draft preparation. **Gábor Mészáros:** conceptualization, data curation, investigation, methodology, supervision, writing – review and editing. **Silvia Bruno:** formal analysis, writing – review and editing. **Elena Ciani:** investigation, methodology, supervision, writing – review and editing. **Johann Sölkner:** conceptualization, funding acquisition, investigation, methodology, supervision, writing – review and editing.

## Funding

This work received no direct funds. The genotyping procedures of the newly produced SNP genotype data were covered by the routine genotyping of ARCHE Austria.

## Ethics Statement

Blood samples from pigs were obtained by specialized professionals following standard breeding procedures and health monitoring practices and guidelines on the farm or at slaughter. No treatments or other procedures with animals were performed that would demand ethical protocols according to Directive 2010/63/EU (2010). Collected DNA or samples from previous projects were also re‐used in this study.

## Conflicts of Interest

The authors declare no conflicts of interest.

## Supporting information


**Figure S1:** Population structure, analysed with a multi‐dimensional scaling (MDS) analysis on a dataset devoid of the commercial breeds (MDS_NC_D) (a), and additionally devoid of the breeds appeared to be close to the AT in the initial MDS analysis (MDS_R_D) (b).

**Figure S2:** Minimum CV error method. The best value of *K* is the one corresponding to the lowest value of CV (a). EVANNO method considers the Δ rate of change between two subsequent values of *K*. The best value of *K* is the first one that shows a Δ rate of change almost equals to zero (b).

**Figure S3:** Analyses performed on ADMIXTURE 1.3.0. Every vertical line refers to an individual and is divided into *K* segment. The segment length is proportional to the genetic component belonging to a specific cluster. Here are shown the main significant *K* values for the balanced/unsupervised analyses (K11—a; K2—b).

**Figure S4:** Mutual Nearest Neighbour Network analyses at *K* = 19 performed with the R package Netview. Each dot represents an individual, whose colour is referred to the belonging population. The k value refers to the maximum number of nearest neighbours that the software infers for every individual.

**Figure S5:** Cluster networks based on FST distances computed removing commercial breeds from GD_I_D.

**Table S1:** Description of the 12 analysed pig breeds. For every breed is specified the origin nation, the number of individuals, and markers in row dataset. Numerical indexes refer to the study/context from which data were collected.
**Table S2:**: Description of all the datasets used in the study. The name, the acronym, the filtering criteria and the intended use are specified for every dataset.
**Table S3:**: Membership coefficients (qi) distribution. The red line signs the threshold under which an individual is defined as admixed (0.8).
**Table S4:**: Pairwise FST and Nei's distances for each analysed breed. The intensity of the colour is proportional to the genetic distance.
**Table S5:**: Genetic migrations obtained with Treemix. Genetic migrations obtained with Treemix. Every migration has an edge weight, whose values range from 100 to 0. This range was arbitrarily divided into 5 windows, represented by a different colour.
**Table S6:**: Treemix f4‐statistics for all the 12 breeds in the dataset. For every pairwised statistic (A–D) is specified: the calculated f4 statistic, which measures allele frequency covariance between (A, B) and (C, D); the standard error of the f4 statistic; the *Z*‐score (f4 statistic/SE), which is used to assess statistical significance.
**Table S7:**: Observed and expected number of homozygotes, number of (nonmissing, non‐monomorphic) autosomal genotype observations (N(NM)), and individual inbreeding coefficient (Fis) have been calculated for every individual in the dataset. Moreover, at the end of every breed, is specified the average inbreeding coefficient ± standard deviation.

## Data Availability

The data generated in this study are included in this published article and in its Supporting Information files. The Croatian Turopolje's, Hungarian Mangalitsa's and Black Slavonian's genome data are available on the article of Lukić et al. (2020) at https://doi.org/10.3389/fgene.2020.00261. The Banija Spotted's genome data were extracted from the study of Zorc et al. (2022) at https://doi.org/10.1186/s12711‐022‐00718‐6. The Swallow bellied Mangalitsa's, Moravka's, and Krškopolje's genome data were extracted from the study of Muñoz et al. (2019). The genome sequencing data referred to the commercial breeds and AT population will be made available for academic purposes after signing a Material Transfer Agreement, in which the owner of the data may impose reasonable restrictions on the use of the data, such as confidentiality or refraining from commercial exploitation.
