# Supplementary material for: Revisiting Genetic Relationships of the Endangered Austrian Turopolje With Balkan and Commercial Pig Breeds Using Genome‐Wide SNP Data
Source: Anim Genet. 2026 May 5;57:e70104. doi: 10.1002/age.70104 (PMC13142207; doi:10.1002/age.70104)
Supplement: Supplementary file 1 — Figure S1: Population structure, analysed with a multi‐dimensional scaling (MDS) analysis on a dataset devoid of the commercial breeds (MDS_NC_D) (a), and additionally devoid of the breeds appeared to be close to the AT in the initial MDS analysis (MDS_R_D) (b). [file AGE-57-0-s002.pdf]

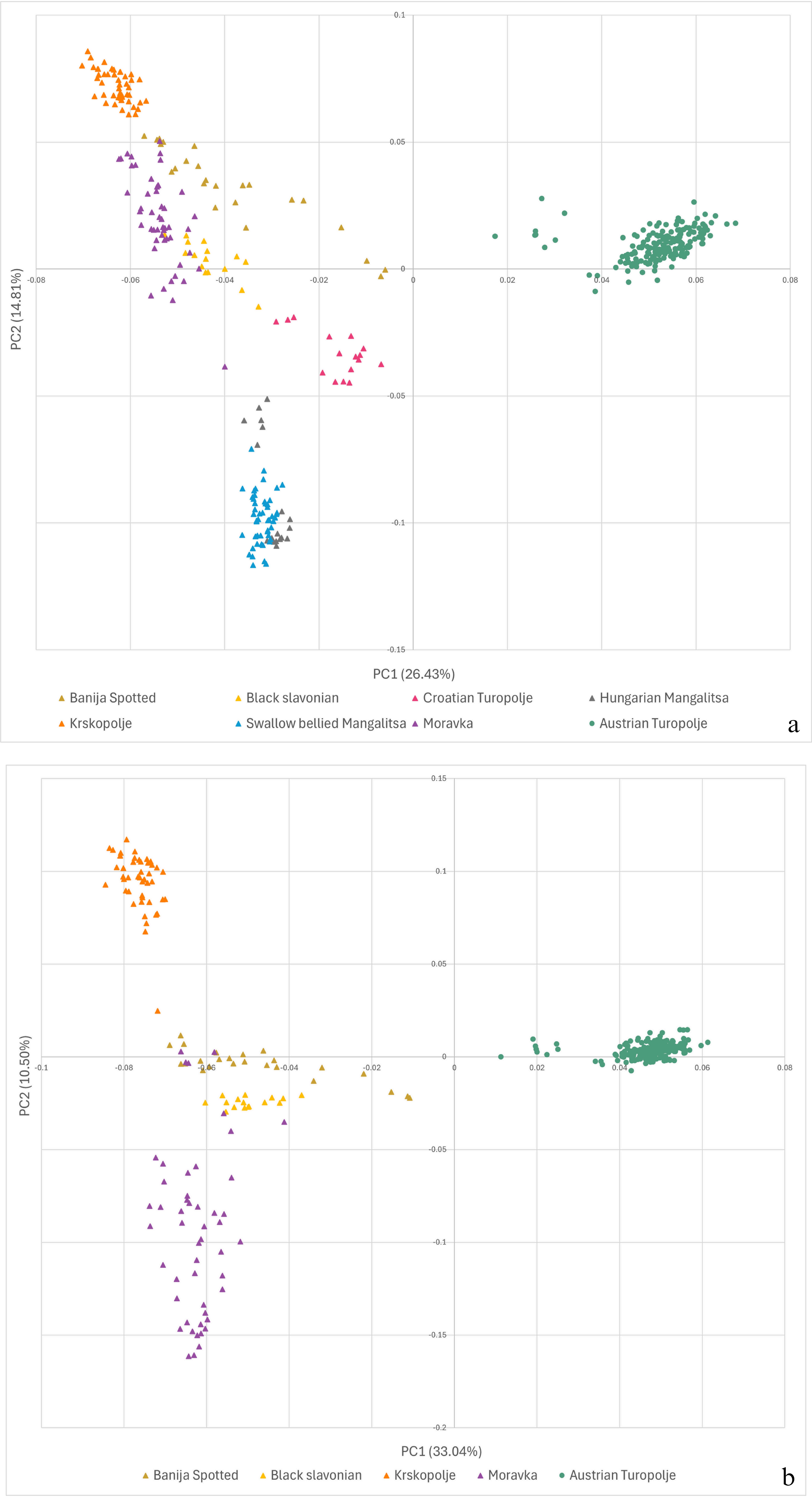

Figure S1: Population structure, analysed with a Multi-Dimensional Scaling (MDS) analysis on a dataset devoid of the commercial breeds (MDS_NC_D) (a), and additionally devoid of the breeds appeared to be close to the AT in the initial MDS analysis (MDS_R_D) (b).
AT population and Balkan breeds are respectively marked with dots and triangles.
