# Supplementary material for: Revisiting Genetic Relationships of the Endangered Austrian Turopolje With Balkan and Commercial Pig Breeds Using Genome‐Wide SNP Data
Source: Anim Genet. 2026 May 5;57:e70104. doi: 10.1002/age.70104 (PMC13142207; doi:10.1002/age.70104)
Supplement: Supplementary file 2 — Figure S2: Minimum CV error method. The best value of K is the one corresponding to the lowest value of CV (a). EVANNO method considers the Δ rate of change between two subsequent values of K. The best value of K is the first one that shows a Δ rate of change almost equals to zero (b). [file AGE-57-0-s003.pdf]

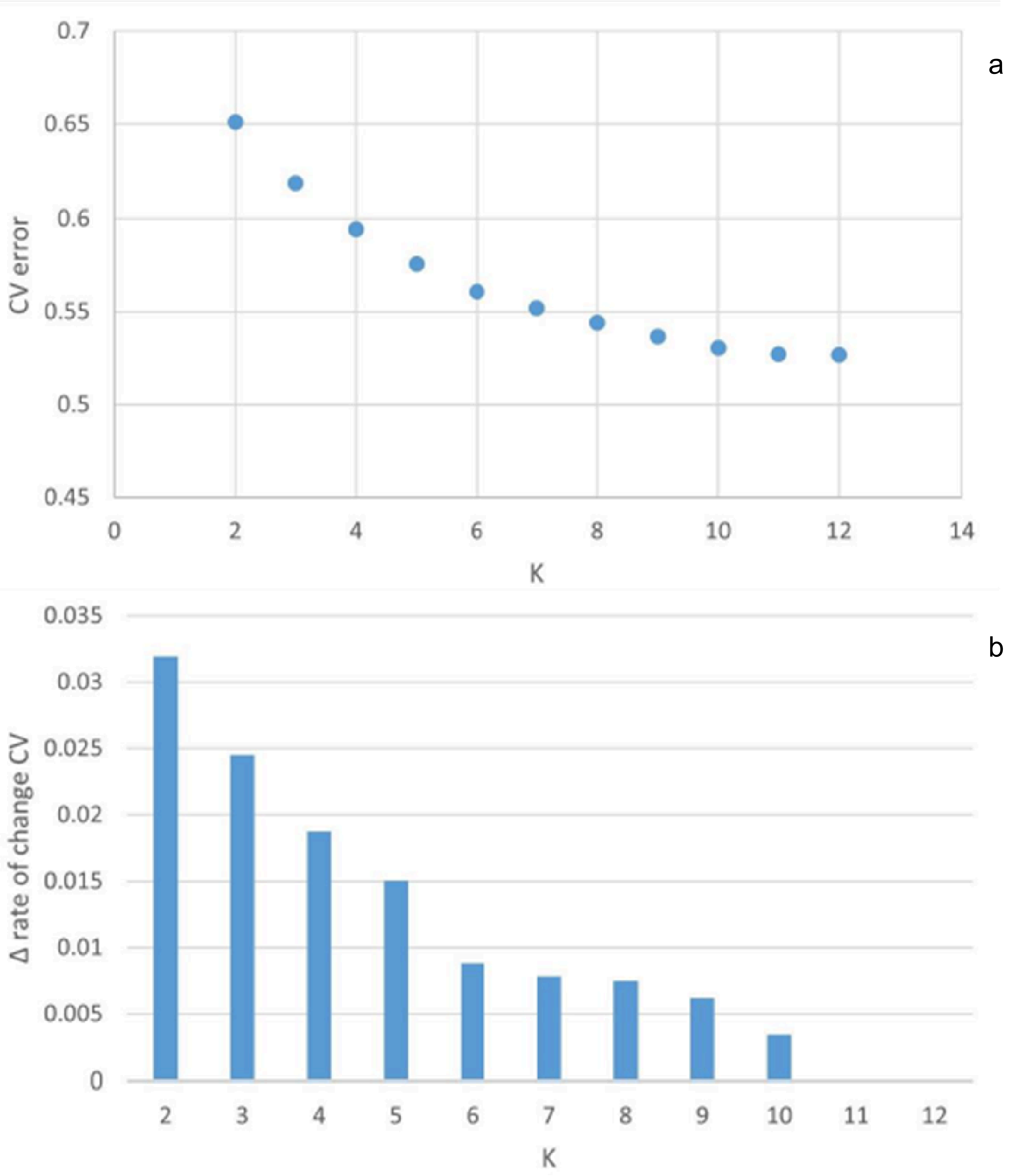

Figure S2. Minimum CV error method. The best value of K is the one corresponding to the lowest value of CV (a). EVANNO method considers the Δ rate of change between two subsequent values of K. The best value of K is the first one that shows a Δ rate of change almost equals to zero (b).
