# Supplementary material for: Revisiting Genetic Relationships of the Endangered Austrian Turopolje With Balkan and Commercial Pig Breeds Using Genome‐Wide SNP Data
Source: Anim Genet. 2026 May 5;57:e70104. doi: 10.1002/age.70104 (PMC13142207; doi:10.1002/age.70104)
Supplement: Supplementary file 3 — Figure S3: Analyses performed on ADMIXTURE 1.3.0. Every vertical line refers to an individual and is divided into K segment. The segment length is proportional to the genetic component belonging to a specific cluster. Here are shown the main significant K values for the balanced/unsupervised analyses (K11—a; K2—b). [file AGE-57-0-s005.pdf]

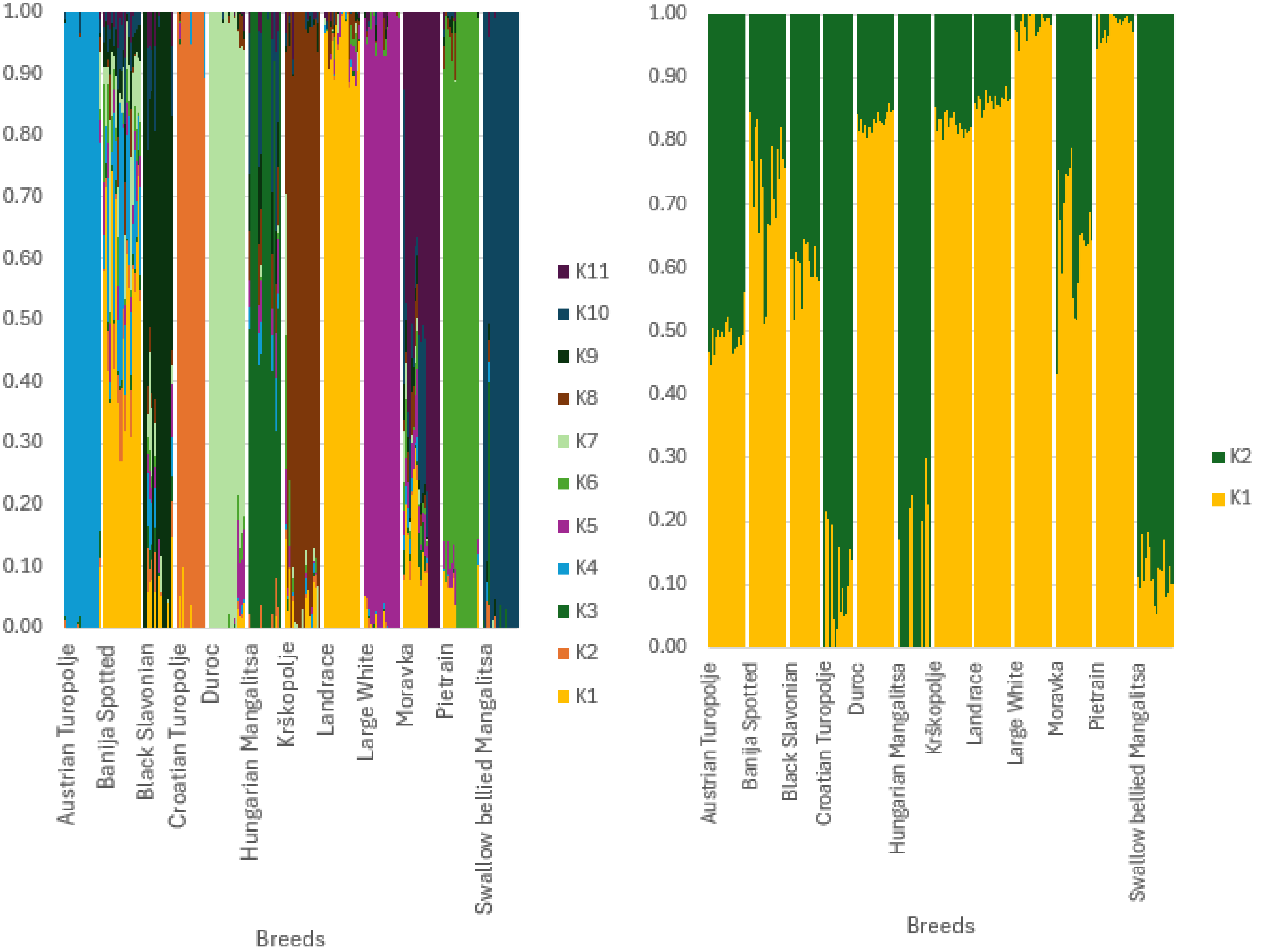

Figure S3. Analyses performed on ADMIXTURE1.3.0. Every vertical line refers to an individual and is divided into K segment. The segment length is proportional to the genetic component belonging to a specific cluster. Here are shown the main significant K values for the balanced/unsupervised analyses (K11 – a; K2 – b).
