# Supplementary material for: Revisiting Genetic Relationships of the Endangered Austrian Turopolje With Balkan and Commercial Pig Breeds Using Genome‐Wide SNP Data
Source: Anim Genet. 2026 May 5;57:e70104. doi: 10.1002/age.70104 (PMC13142207; doi:10.1002/age.70104)
Supplement: Supplementary file 4 — Figure S4: Mutual Nearest Neighbour Network analyses at K = 19 performed with the R package Netview. Each dot represents an individual, whose colour is referred to the belonging population. The k value refers to the maximum number of nearest neighbours that the software infers for every individual. [file AGE-57-0-s001.pdf]

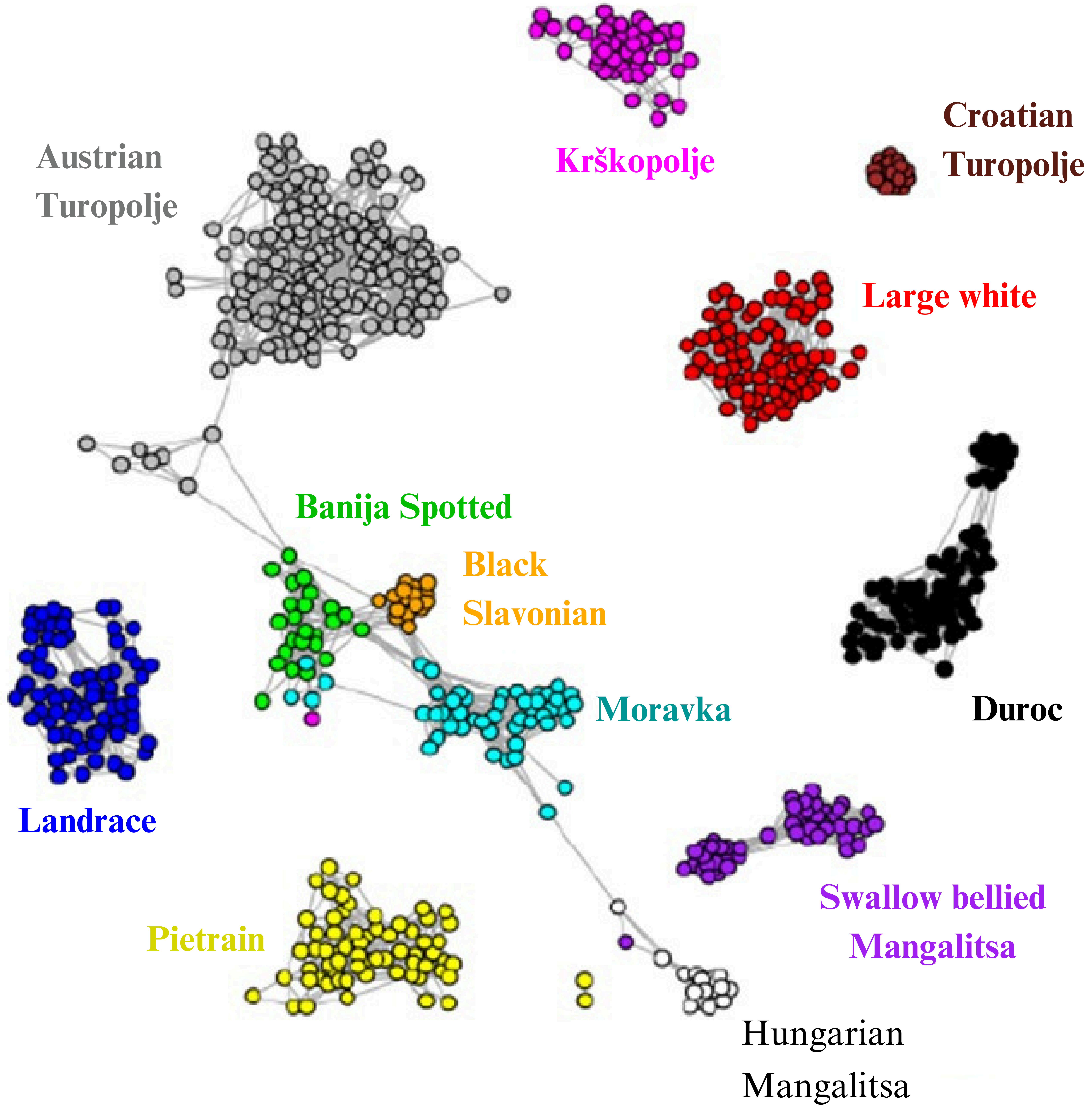

Figure S4. Mutual Nearest Neighbour Network analyses at K=19 performed with the R package Netview. Each dot represents an individual, whose colour is referred to the belonging population. The k value refers to the maximum number of nearest neighbours that the software infers for every individual.
