# Supplementary material for: Revisiting Genetic Relationships of the Endangered Austrian Turopolje With Balkan and Commercial Pig Breeds Using Genome‐Wide SNP Data
Source: Anim Genet. 2026 May 5;57:e70104. doi: 10.1002/age.70104 (PMC13142207; doi:10.1002/age.70104)
Supplement: Supplementary file 5 — Figure S5: Cluster networks based on FST distances computed removing commercial breeds from GD_I_D. [file AGE-57-0-s004.pdf]

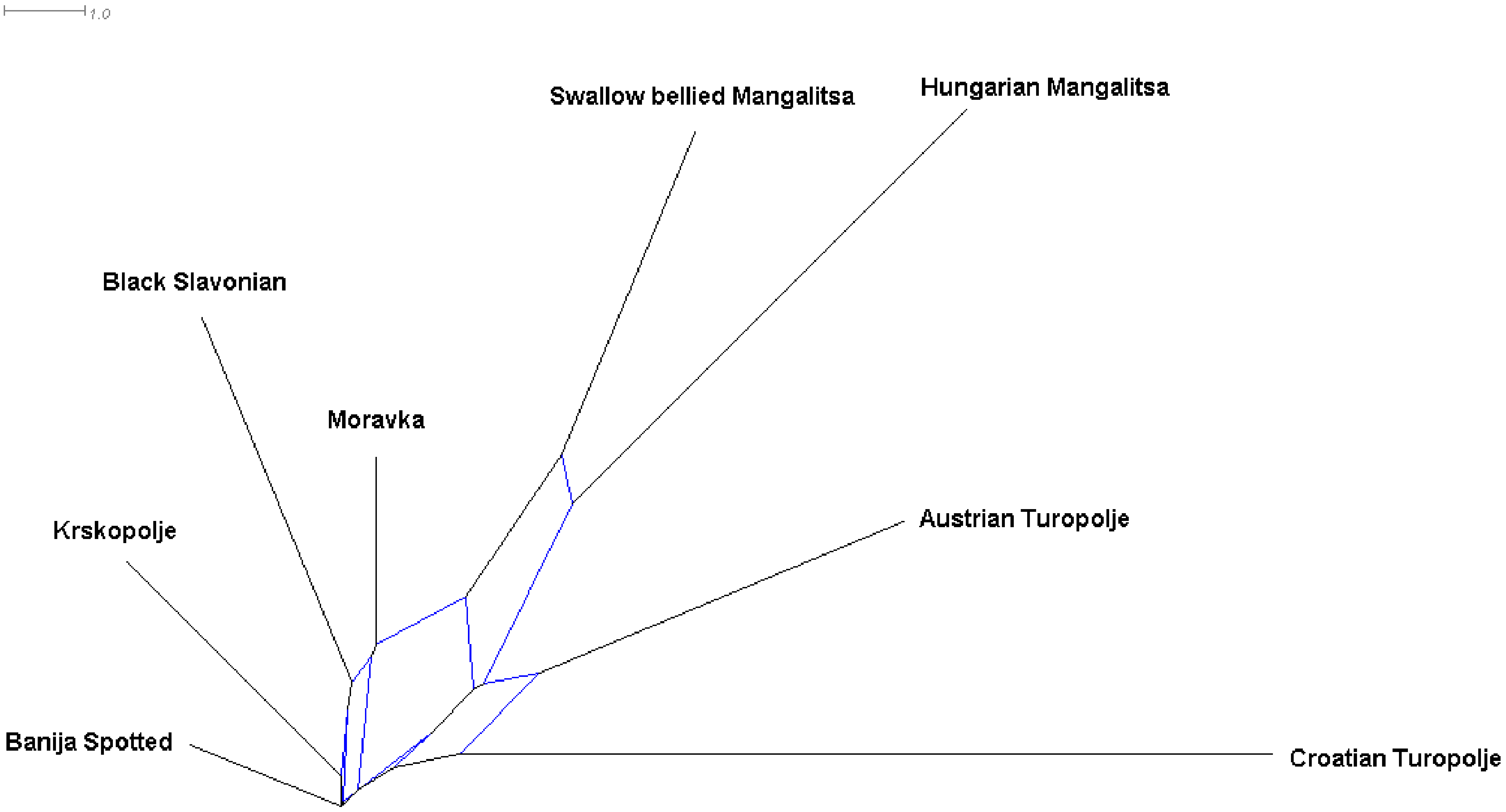

Figure S5: Cluster networks based on FST distances computed removing commercial breeds from GD_I_D.
